# Investigation of Cigarette Smoking among Male Schizophrenia Patients

**DOI:** 10.1371/journal.pone.0071343

**Published:** 2013-08-15

**Authors:** Jundong Jiang, Yuen Mei See, Mythily Subramaniam, Jimmy Lee

**Affiliations:** 1 Research Division, Institute of Mental Health/Woodbridge Hospital, Singapore, Singapore; 2 Saw Swee Hock School of Public Health, National University of Singapore, Singapore, Singapore; 3 Department of General Psychiatry 1, Institute of Mental Health/Woodbridge Hospital, Singapore, Singapore; 4 Duke-NUS Graduate Medical School, National University of Singapore, Singapore, Singapore; Charité-Universitätsmedizin Berlin, Germany

## Abstract

Male schizophrenia patients are known to have a heavier smoking pattern compared with the general population. However, the mechanism for this association is not known, though hypothesis that smoking could alleviate symptomatology of schizophrenia and reduce side effects of antipsychotics has been suggested. The aims of this study were to validate the heavier smoking pattern among male schizophrenia patients and to investigate the possible mechanisms for the association. To enhance the reliability of the study, we recruited two large independent samples with 604 and 535 male Chinese schizophrenia patients, and compared their smoking pattern with that of 535 healthy male controls recruited from general population. Validated multiple indicators and multiple causes structure equation model and regression models were used to investigate the association of smoking with factors of schizophrenia symptomatology and with the usage of antipsychotics and their extra-pyramidal side effects (EPS). Schizophrenia patients had significantly heavier smoking pattern compared with healthy controls in our sample (42.4% vs. 16.8%, p<0.001 for current smoking prevalence; 23.5% vs. 43.3%, p<0.001 for smoking cessation rate; 24.5% vs. 3.0%, p<0.001 for heavy smoker proportion). Their smoking status was also found to be consistently and significantly associated with reduced negative factor scores for schizophrenia symptomatology (β = −0.123, p = 0.051 for sample-A; β = −0.103, p = 0.035 for sample-B; β = −0.082, p = 0.017 for the combined sample). However, no significant association was found between smoking and antipsychotics usage or risk of EPS. These results support that smoking is associated with improved negative symptoms, which could account for the heavier smoking pattern among schizophrenia patients.

## Introduction

Schizophrenia patients have been widely reported to have heavier smoking pattern when compared with general population and patients with other mental disorders. De Leon et al. [1] reviewed 42 studies across 20 nations and found that the current smoking rate among male schizophrenia patients was constantly higher than that of general population. Numerous studies have also found that schizophrenia patients tend to consume higher tar and higher nicotine containing cigarettes and to extract more nicotine from smoking [2]–[4]. In addition, cessation rate of smoking was also found to be significantly lower among male schizophrenia patients [5]–[7]. This heavier smoking pattern may impose a significant burden on patients with schizophrenia since 20% of reduction in life expectancy among patients with psychotic disorders is thought to be contributed by smoking [8].

The reason for the heavy smoking pattern among schizophrenia patients is not known. One widely believed explanation was that smoking could reduce the side effects of antipsychotics [9], [10]. Antipsychotics act by blocking dopamine receptors and can trigger various side effects including extrapyramidal side effects (EPS). Nicotine in cigarette could induce dopamine release in pre-frontal cortex and could also increase hepatic clearance of antipsychotics by activating cytochrome P450 enzymes [11]. As such, the EPS of antipsychotics, which are expressed as involuntary movement symptoms, are believed to be alleviated by smoking. Studies showing that schizophrenia patients with high dose of antipsychotics tended to be heavier smokers appear to support this explanation [12]–[14]. However, studies that directly examine the effects of smoking on the EPS of antipsychotics (e.g., dyskinesia) yield conflicting results [7], , suggesting that this hypothesis needs further studies.

Another hypothesis for heavy smoking pattern among schizophrenia patients is that smoking could alleviate schizophrenia symptomatology, particularly negative symptoms, by improving the sensory gating deficit and raising dopamine levels in brain [16], [17]. Experimental and observational studies have provided support for this explanation by finding an association between heavier smoking and improved negative symptoms [18], [19]. However, evidence on the association is not consistent since insignificant findings have also been reported [20]–[22]. Small sample size might contribute to the insignificant findings as the studies may lack the power to detect the difference if effects of smoking are small. In addition, the subscale scores (i.e., positive, negative, and general psychopathology) of the Positive and Negative Syndrome Scale (PANSS) are conventionally used to represent the severity of symptoms. However, these three subscales effectively represent a mixture of dimensions of symptoms since PANSS has been found to measure up to 5 dimensions of symptomatology [23]. Since smoking may not have the same effects on all the factors of the symptoms, not taking the structure of PANSS into account may also affect the findings on the association of smoking and symptomatology.

The aims of this study were to examine the smoking pattern among male Chinese schizophrenia patients in Singapore. We also attempted to investigate the possible mechanisms for their heavy smoking pattern by examining the association of smoking with symptomatology of schizophrenia and with antipsychotics usage and their side effects. To enhance the reliability of the study, two independent study samples of male schizophrenia patients were recruited. We have previously reported a validated 5-factor structure model of the PANSS [24], which we applied to examine the association of smoking with the symptom factors of schizophrenia. The findings of this study could reveal the smoking pattern among male schizophrenia patients and could also help in understanding the mechanism for persistent smoking among them.

## Methods

### Ethics Statement

This study, including the consent process, was approved by the National Healthcare Group Domain Specific Review Board of Singapore. Written informed consent was obtained from all study participants who were recruited into the study before any assessments. Participation was voluntary and participants were allowed to reject or withdraw at any point with no disadvantage to their treatments.

### Sample Recruitments

Male patients with schizophrenia were recruited from the Institute of Mental Health (IMH) in Singapore under the Singapore Translational & Clinical Research in Psychosis (STCRP) program. Two independent study samples were recruited under this project: Sample-A was recruited between 2005 and 2008 and consisted of 604 male patients with schizophrenia; Sample-B was recruited between 2008 and 2012 and consisted of 535 male patients. In both samples, patients were included if they were of Chinese ethnicity and met DSM-IV-TR diagnostic criteria for schizophrenia, and if they consented to participate in our study. Patients were excluded from the study if they had organic brain causes for psychosis, mental retardation or a current substance use disorder. A total of 535 healthy male controls were also recruited from the community in Singapore between 2008 and 2012 to serve as a reference comparison group. The controls were included into the study if they were of Chinese ethnicity, and they were excluded from the study if they had a history of psychiatric disorders, except for nicotine related disorders.

### Measures and Covariates

All participants were assessed with the Structured Clinical Interview for DSM-IV-TR (SCID) and PANSS by clinicians and research psychologists, who were trained on the administration of the instruments. Demographic and smoking information were collected during an interview. Both patients and controls were classified as current-smoker if they smoked at least one cigarette per day at the point of assessment. They were classified as ex-smoker if they had smoked in the past but had quit smoking at the time of assessment. Ever-smoker was defined as those who were either current- or ex-smokers. Among the current smokers, subjects were classified as light smoker if they smoked 10 cigarettes or less per day and were classified as heavy smoker if they smoked more than 10 cigarettes per day. The medication profile of schizophrenia patients were collected from hospital records and the total daily antipsychotics dose was computed by summation of the daily chlorpromazine equivalent dose of all prescribed antipsychotics [25]. The EPS of antipsychotics were assessed by two instruments: Abnormal Involuntary Movement Scale (AIMS) and Simpson-Angus Scale (SAS) instruments [26], [27]. Based on AIMS score, patients were classified as tardive dyskinesia, which is a chronic movement disorder characterized by repetitive, involuntary and purposeless movements, according to the Schooler and Kane criteria [28]. Based on SAS score, patients were classified to have drug-induced Parkinsonism, a syndrome characterized by extrapyramidal rigidity, tremor at rest, and postural instability, if the score is more or equal to 3.

### Statistical Analysis

In comparing the smoking pattern between patients with schizophrenia and healthy controls, Chi-squared test was used in assessing the significance for nominal and ordinal outcomes and t-test with unequal variance was used to assess the significance for continuous outcomes. Adjusted p values for smoking rate were computed by logistic regression to control for covariates including age, education, occupation and living apartment type, which might have effects on smoking pattern.

In elucidating the association of smoking with symptomatology of schizophrenia, a multiple indicators and multiple causes (MIMIC) structure equation model was used. The model was derived based on the validated factor model of PANSS identified in our previous study [24]. In the model, the 30 items of PANSS were deconstructed into 5 latent factors: positive factor, negative factor, depression factor, excitement factor, and cognitive factor. Each factor was measured by a subset of the 30 items, and represented a distinct dimension of schizophrenia symptoms. Each of the five factors was hypothesized to depend on current smoking status (i.e., non-smoker, light smokers and heavy smokers) and antipsychotics dose, which had a major effect in influencing current symptoms. The effect size of smoking on symptomatology after controlling for antipsychotics usage could be assessed by its loading and significance on the five latent factors. To achieve reliability, we applied the model on the two independent datasets (i.e., Sample-A and Sample-B) separately and also on the combined dataset to examine the consistency of the effects of smoking. In elucidating the association of smoking with antipsychotics usage and its EPS, general linear model with log link (i.e., for antipsychotic dose) or logit link (i.e., for tardive dyskinesia and drug-induced Parkinsonism) was used to accommodate the distribution of the dependent variables (i.e., antipsychotics dose distribution was positively skewed; tardive dyskinesia or drug-induced Parkinsonism was binomially distributed).

Comparisons of smoking pattern were performed under STATA 10.0, and examination of the association of smoking with the factors of schizophrenia symptomatology was performed under Mplus 6.11.

## Results

### Descriptive Statistics of Study Samples

Descriptive statistics of the three study samples are showed in Table 1. Healthy controls were younger than patients from both samples, and patients from Sample-B were younger than patients from Sample-A. Patients had significantly lower socio-economic status than healthy controls (i.e., lower education, smaller apartments and higher unemployment rate). About one quarter (25.5%) of patients in Sample-A had tardive dyskinesia disorder and about one third (32.1%) of them had drug-induced Parkinsonism.

**Table 1:** Descriptive statistics of the three independent study samples.

	Sample-A Cases^1^N = 604	Sample-B Cases^2^N = 535	Healthy ControlsN = 535	Nominal p value^3^
**Age (yrs)**	49.2±13.1	41.8±10.9	33.1±10.2	<0.001
**Antipsychotics dose (CPZ equivalents, mg)**	491.5±527.1	424.5±433.5	–	
**PANSS**				
Positive subscale	9.91±3.79	11.8±5.74	–	
Negative subscale	12.3±5.27	12.9±6.31	–	
General psychopathology subscale	21.3±5.27	24.1±8.77	–	
**Tardive dyskinesia percentage**	25.5%	–	–	
**Drug-induced Parkinsonism percentage**	32.1%	–	–	
**Education**				<0.001
Less than Primary^4^	–	0.39%	0.00%	
Primary	–	19.3%	1.33%	
Secondary	–	49.4%	25.3%	
Junior College or polytechnic	–	19.3%	27.6%	
University or higher	–	11.6%	45.7%	
**Living Apartment**	–			<0.001
1-room	–	8.32%	0.23%	
2/3-room	–	31.3%	15.2%	
4/5-room	–	41.0%	64.8%	
Private house	–	3.46%	12.1%	
Others^4^	–	15.5%	7.69%	
**Occupation**				<0.001
Unemployed	–	55.7%	7.94%	
Manual labor	–	4.47%	0.95%	
Admin and sales	–	3.89%	8.32%	
Professional management	–	0.78%	14.5%	
Others^4^	–	35.2%	68.2%	

1 Education, living apartment and occupation information were not collected for sample-A.

2 AIMS and SAS assessments were only performed on patients in Sample-A. Therefore, tardive dyskinesia and drug-induced Parkinsonism status were not available for Sample-B.

3 Nominal p values were computed based on Sample-B cases and healthy controls.

4 The group was used as reference group in regression analysis.

### Smoking Pattern of Schizophrenia Patients

As shown in Table 2, the prevalence of smoking in patients was significantly higher than that of healthy controls (54.1% vs. 29.3% for life time prevalence, RR = 1.83, p<0.001; 42.4% vs. 16.8% for current prevalence, RR = 2.52, p<0.001; RR stands for relative risk). The current cessation rate, which measures the proportion of smokers who had quit smoking by the time of assessment, was also significantly lower for schizophrenia patients (23.5% vs. 43.3%, RR = 0.54, p<0.001). Both current smoking rate and cessation rate remained to be highly significant even after controlling for age, education, occupation, and living apartment (p = 0.002 for current smoking rate and p = 0.004 for cessation rate). All smoking metrics between Sample-A and Sample-B were not significantly different (p value = 0.126, 0.707, 0.290, 0.277 for current-smoker, ex-smoker, ever-smoker and cessation rate, respectively), though patients from Sample-A had slightly lower smoking prevalence and higher quitting rate.

**Table 2:** Comparison of smoking patterns between schizophrenia male patients and male healthy controls.

	Sample-AN = 604	Sample-BN = 535	Healthy controlsN = 535	Risk Ratio (RR)/difference^1^	P value^1^	Adjusted p value^2^
**Ever smoker**	50.9%	54.1%	29.5%	1.83	<0.001	0.129
**Current smoker**	37.9%	42.4%	16.8%	2.52	<0.001	0.002
**Ex-smoker**	13.4%	12.7%	12.8%	1.00	0.957	0.143
**Cessation rate**	26.9%	23.5%	43.3%	0.54	<0.001	0.004
**Current smoking status**					<0.001	<0.001
Non-smoker	62.1%	55.3%	82.6%			
Light smoker	13.4%	14.4%	11.6%			
Heavy smoker	13.9%	24.5%	3.00%			
Missing	10.6%	5.79%	2.80%			
**Smoking starting age** 3	–	24.1	24.0	0.1	0.920	0.042
**Smoking starting age w.r.t** **onset of psychosis (yrs)** 3	–	0.045	–	–	0.951	–

1 Measures were obtained based on Sample-B cases and healthy controls.

2 Adjusted p values were computed after controlling for age, education, living apartments and occupation.

3 Smoking starting age information was not collected for sample-A.

### Association of Smoking with Antipsychotics Usage and their Extra-pyramidal Side Effects

As shown in Table 3, the association of smoking with antipsychotics dose was inconsistent and insignificant after adjusting for symptoms severity (p = 0.182, 0.058, 0.740, for sample-A, sample-B, and combined sample). No significant association was found between smoking and tardive dyskinesia or drug-induced Parkinsonism, which are due to EPS of antipsychotics.

**Table 3:** Association of smoking with antipsychotics dose and EPS in regression analysis.

Dependent variables	Samples	Smoking effect size (β)	Nominal p value	Adjusted p value^1^
Antipsychotics dose(CPZ equivalent)	Sample-A	−0.081	0.171	0.182
	Sample-B	0.094	0.035	0.058
	Combined	−0.013	0.939	0.740
Tardive dyskinesia^2^	Sample-A	−0.067	0.100	0.668
Drug-induced Parkinsonism^2^	Sample-A	−0.068	0.172	0.624

1 Severity of symptoms, total positive symptoms score, total negative symptom score, and total general symptoms score were adjusted as covariates for association study of antipsychotic dose, and duration of illness were adjusted as covariates for association study of EPS.

2 AIMS and SAS assessments were only performed on patients in Sample-A. Therefore, tardive dyskinesia and drug-induced Parkinsonism status were not available for Sample-B.

### Association of Smoking with Symptomatology of Schizophrenia

**Figure 1:**
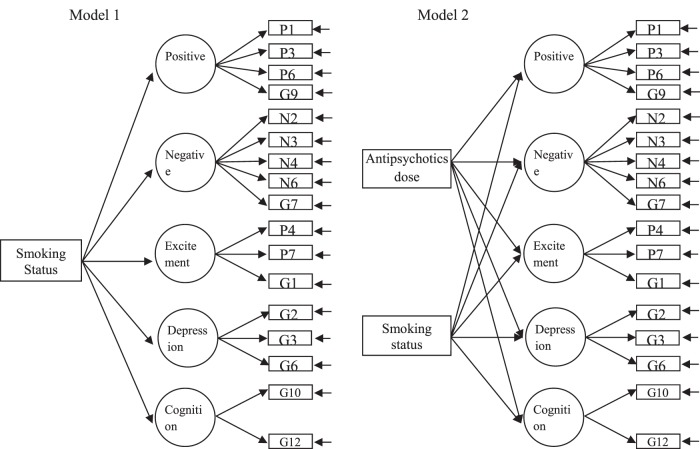
MIMIC model in assessing the effects of current smoking status on symptomatology of schizophrenia.

The association of smoking on symptomatology were assessed based on the MIMIC structural equation model as depicted in Figure 1. The 30 items of PANSS were deconstructed into 5 factors based on our previous validated factor analysis of PANSS [24]: Positive factor was measured by P1delusions, P3 hallucinations, P6 suspiciousness and G9 unusual thought content; negative factor was measured by N2 emotional withdrawal, N3 poor rapport, N4 social withdrawal, N6 lack of spontaneity and G7 motor retardation; excitement factor was measured by P4 excitement, P7 hostility, G14 poor impulse control; depression factor was measured by G2 anxiety, G3 guilt feelings and G6 depression; cognitive factor was measured by G10 disorientation and G12 lack of judgement and insight. Two MIMIC models were built to assess the effects of smoking status. In model 1, current smoking status was hypothesized as the only factor that could affect symptomatology. In model 2, we further hypothesized that antipsychotic dose could also affect current symptoms of schizophrenia. Both models could fit the two independent study samples as well as the combined sample well (CFI>0.9, RMSEA<0.08, Table S1).

As shown in Table 4, negative dimension of symptomatology was the only factor that was consistently and significantly reduced by smoking (β = −0.125, −0.096, −0.080; p value = 0.047, 0.052, 0.021 for Sample-A, Sample-B and combined sample, respectively). Statistical significance remained even after controlling for antipsychotic usage (β = −0.123, −0.103, −0.082; p value = 0.051, 0.035, 0.017 for Sample-A, Sample-B and combined sample, respectively). Smoking seemed to increase the excitement dimension of the symptoms (β  = 0.085, p<0.05 for the combined sample), but it did not reach significance in the individual study samples.

**Table 4:** Association of smoking with the factors of PANSS.

variable	Latent Factors	Sample-A		Sample-B		Combined	
		β^1^	P value	β^1^	P value	β^1^	P value
Model 1: Smoking status	Positive	0.024	0.723	0.031	0.595	0.062	0.153
	**Negative**	−**0.125**	**0.047**	−**0.096**	**0.052**	−**0.080**	**0.021**
	Excitement	0.068	0.378	0.050	0.235	0.085	0.029
	Depression	0.118	0.065	−0.029	0.490	0.057	0.096
	Cognitive	−0.253	0.003	0.127	0.006	0.042	0.254
Model 2: Smoking status	Positive	0.038	0.577	0.023	0.692	0.068	0.119
	**Negative**	−**0.123**	**0.051**	−**0.103**	**0.035**	−**0.082**	**0.017**
	Excitement	0.077	0.335	0.048	0.243	0.085	0.028
	Depression	0.127	0.053	−0.036	0.395	0.058	0.097
	Cognitive	−0.248	0.004	0.126	0.006	0.043	0.251

1 β is the factor loading of smoking status on the five latent factors of symptomatology of schizophrenia in the MIMIC model.

## Discussion

### Smoking Pattern of Schizophrenia Patients

More than half of male Chinese patients with schizophrenia had smoked in their life time, and 40.0% of them were current smokers in our study (i.e., combined sample). Although these prevalence estimates are significantly higher than that of healthy controls in our samples, they are smaller than the prevalence in studies conducted in other countries. De Leon & Diaz et al. [1] found that the average prevalence of current smokers among male schizophrenia patients was 72% based on 14 studies conducted in 8 countries. A recent Chinese study has also shown that 76% male schizophrenia patients were smokers in China [7]. The apparent lower smoking prevalence in our study might be attributed to the effective anti-smoking campaigns in Singapore (i.e., Health Promotion Board, Singapore) [29], [30]. This is because the smoking prevalence among the male healthy controls in our study (i.e., 16.8%) is also much lower than that in other countries (i.e., the smoking prevalence was 53% among healthy male controls in the 14 studies reviewed by de Leon et al. [1]). In our study, we also found that the cessation rate among male schizophrenia patients was only 0.54 of that among males in general population, suggesting that schizophrenia patients have greater difficulty in quitting smoking despite the anti-smoking campaigns in Singapore. This observation is also in agreement with previous findings that schizophrenia patients had significantly lower cessation rate than general population [6], [7], [30].

### Association of Smoking with Antipsychotics Usage and EPS

It is suggested that smoking could enhance dopamine release and metabolism of antipsychotics to reduce their EPS, which has been hypothesized to be one of the reasons for the heavier smoking pattern among schizophrenia patients [10], [31], [32]. Observations that patients with schizophrenia who received higher dose of antipsychotics are more likely to be smokers appear to support this hypothesis since they are exposed to more EPS [12]–[14]. However, in our samples, the association of smoking with antipsychotic dose was neither consistent nor significant. While smokers appeared to be associated with higher dose of antipsychotics in sample-B, the direction of association was reversed in sample-A, and the associations were not statistically significant. Moreover, we did not find any association between smoking and tardive dyskinesia or drug-induced Parkinsonism, which are due to EPS of antipsychotics. Therefore, our results do not support the association of smoking with antipsychotic dose or antipsychotic EPS.

### Associations of Smoking with Schizophrenia Symptomatology

Smoking was found to be associated with reduced negative factor of symptoms of schizophrenia, which is measured by N2, N3, N4, N6, and G7 of PANSS in our dataset. However, no significant effects of smoking were found on the positive factor measured by P1, P3, P6 and G9. This observation is consistent with the findings by Smith et al. [18], who conducted a double blinded randomized control trial on male schizophrenia patients and found that smoking could significantly reduce negative symptoms but not positive symptoms. Our observation is also in agreement with findings from two recent studies in China that current male smokers had milder negative symptoms than non-smokers among male schizophrenia patients [7], [33]. Similar observations of improvement in negative symptoms among smokers have also been reported in Caucasian population and Egyptian population [19], [34]. Taking the evidence together, our results support the hypothesis that smoking could improve the negative symptoms of schizophrenia, which could possibly lead to the heavier smoking pattern among schizophrenia patients [35], [36]. The excitement dimension of schizophrenia symptomatology also seemed to be increased by smoking. However, the effect size was not significant in the individual samples.

### Strengths and Limitations

The main strength of this study is that two large independent study samples were employed to explore the smoking pattern and the possible mechanisms for the heavier smoking pattern among male schizophrenia patients was also examined. The comparability of the smoking pattern and the consistency of the smoking effects on negative factors across the two studies suggest that our findings are reliable. In addition, we utilised a validated PANSS factor structure [24], which make the investigation of smoking on underlying dimensions of the symptomatology of schizophrenia feasible. However, this study is also limited by the cross sectional nature of the sample recruitment. Therefore, although we found a significant and consistent association between smoking and improved negative symptoms of schizophrenia, it is still impossible to prove definitively that the reduction in negative symptoms is due to smoking. However, based on prior knowledge, especially the experimentally controlled study conducted by Smith et al. [18], we believe it is plausible that smoking improved the negative symptoms.

### Conclusion

In conclusion, our results showed that male Chinese patients with schizophrenia had significantly higher smoking and lower cessation rates than general population. We found that smoking is consistently and significantly associated with improved negative symptoms, while no significant association was found between smoking and antipsychotics and their EPS. These observations support the hypothesis that smoking alleviates negative symptoms in schizophrenia patients, which may account for the heavier smoking pattern among schizophrenia patients. Although smoking has a wide range of well-established ill effects on human health, these findings do raise the possibility of exploring nicotinic pathways for novel treatments of schizophrenia. Indeed, transdermal nicotine treatment on non-smoking schizophrenia patients was shown to significantly improve short term cognitive function [37]. Recent trials with nicotinic receptor agonists have also shown promise in alleviating cognitive impairments and negative symptoms in schizophrenia [38], [39].

## Supporting Information

Table S1 The fittings of the MIMIC models on the two independent and the combined study samples.
(DOCX)
