# Supplementary material for: Investigation of Cigarette Smoking among Male Schizophrenia Patients
Source: PLoS One. 2013 Aug 15;8(8):e71343. doi: 10.1371/journal.pone.0071343 (PMC3744579; doi:10.1371/journal.pone.0071343)
Supplement: Table S1 — The fittings of the MIMIC models on the two independent and the combined study samples. (DOCX) [file pone.0071343.s001.docx]

**Table S1.** The fittings of the MIMIC models on the two independent and the combined study samples.

| **Study Samples** | **RMSEA** | | **CFI** | | **NNFI** | |
| --- | --- | --- | --- | --- | --- | --- |
|  | Model 1 | Model 2 | Model 1 | Model 2 | Model 1 | Model 2 |
| Sample-A | 0.049 | 0.047 | 0.979 | 0.980 | 0.973 | 0.974 |
| Sample-B | 0.073 | 0.071 | 0.914 | 0.910 | 0.891 | 0.886 |
| Combined | 0.058 | 0.057 | 0.953 | 0.951 | 0.940 | 0.937 |
